# 

**Published:** 1914-01-10

**Authors:** 


### Appointments.

Dr. F. M. Loughlane, M.R.C.S. (Eng.), L.R.C.P. (Lond.), has been appointed third assistant medical officer at Camberwell Infirmary. He is at present casualty officer and resident anæsthetist at St. Thomas's Hospital, an appointment which he has held since August last. Previously he was house surgeon at the hospital, and prior to that had been house surgeon at Bangor Infirmary.

Dr. R. F. C. Talbot has been appointed resident medical officer to Barrasford Sanatorium, Northumberland. Mr. Talbot, who has been in the Indian Medical Service, graduated M.B., B.S. at Dublin University in 1900. is also a Fellow of the Royal College of Surgeons, Ireland, and succeeds Dr. W. C. Rivers, who, as previously announced in The Hospital, has been appointed tuberculosis officer to the West Riding of Yorkshire.

Rates of Subscription: Twelvemonths, 6/6; Six months, 4/-; Three months, 2/-, post free to any address in the United Kingdom. Abroad, Twelvemonths, 8/8; Six months, 5/-; Three months, 2/6, post free.—Address The Subscription Dept., "The Hospital," The Hospital Building, 8/292 Southampton Street, Strand, London, W.C.

